# Supplementary material for: GALBA: Genome Annotation with Miniprot and AUGUSTUS
Source: bioRxiv. 2023 Apr 10:2023.04.10.536199. Preprint. [Version 1] doi: 10.1101/2023.04.10.536199 (PMC10120627; doi:10.1101/2023.04.10.536199)
Supplement: Supplement 1 [file NIHPP2023.04.10.536199v1-supplement-1.pdf]

bioRxiv preprint doi: https://doi.org/10.1101/2023.04.10.536199; this version posted April 10, 2023. The copyright holder for this preprint (which was not certified by peer review) is the author/funder, who has granted bioRxiv a license to display the preprint in perpetuity. It is made available under aCC-BY 4.0 International license.

Supplementary Materials

Supplementary Figures

```
##ATN GAGGCC---CGCTCACCgtactgactgatgccatcggtatcgattcggagctagcttagtcaagCACAAGCGCTATAGCCTAC
##ATA E..A..-..R..S..P.                                               .T..$$R..Y..!A..Y..
##AAS |  |           |                                                |    |  +   |  |
##AQA E  A  F  H  -  P                                                T  E R  W   A  Y
```

Figure S1: Custom alignment format produced by miniprot executed with option `--aln`. Here, `ATN` stands for target nucleotides, `ATA` for translated target codons, `AAS` for amino acid alignment quality, and `AQA` for query protein amino acids. “$” and “!” represent frameshifts. If an intron is longer than 200bp, only 100+100bp are shown while an integer in the middle may indicate the total intron length, e.g.: `...gtcatgcta~500~tacgatgactag....`

bioRxiv preprint doi: https://doi.org/10.1101/2023.04.10.536199; this version posted April 10, 2023. The copyright holder for this preprint (which was not certified by peer review) is the author/funder, who has granted bioRxiv a license to display the preprint in perpetuity. It is made available under aCC-BY 4.0 International license.

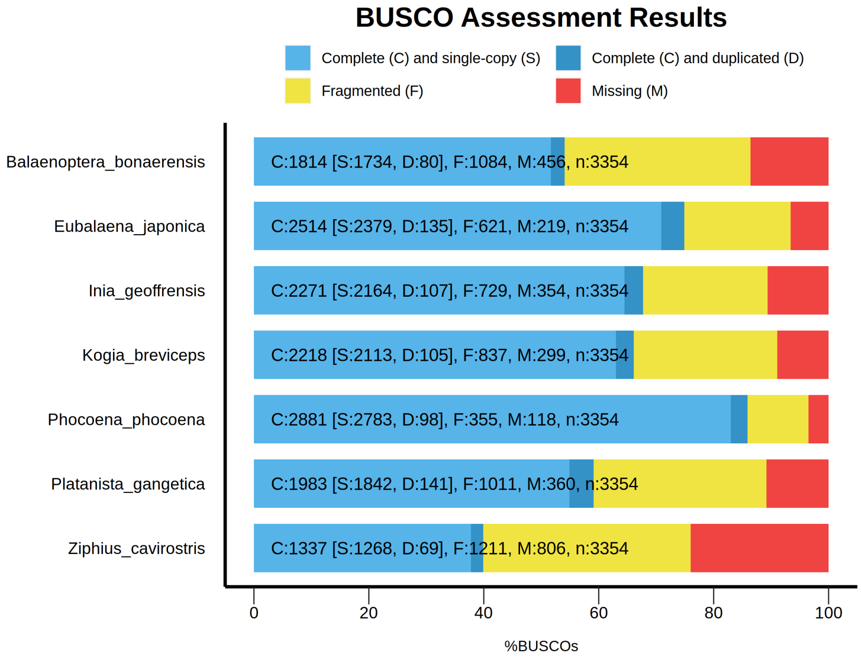

Figure S2: BUSCO scores (obtained with vertebrata_odb10) in whale and dolphin genome assemblies.

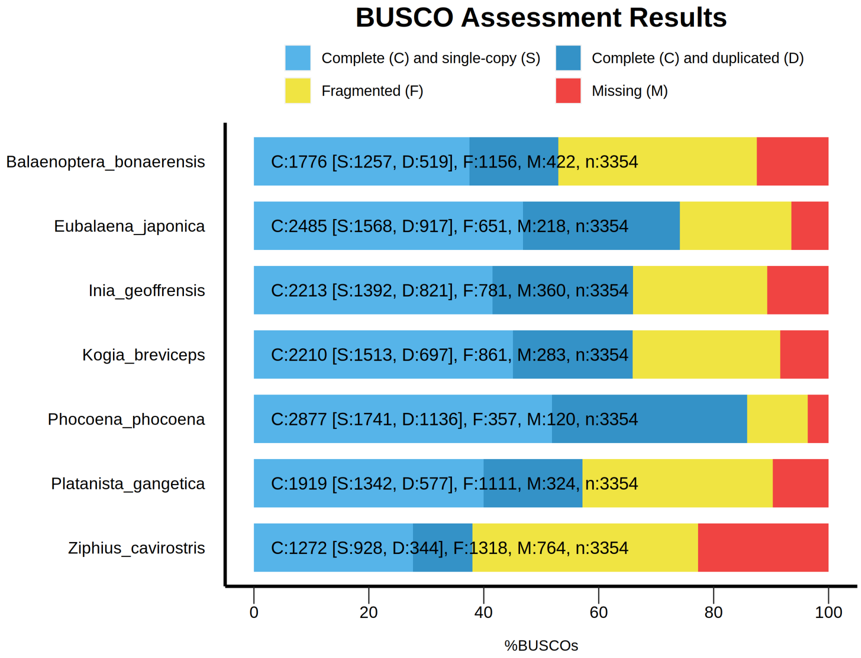

Figure S3: BUSCO scores (obtained with vertebrata_odb10) of proteins predicted with GALBA in whale and dolphin genomes.

bioRxiv preprint doi: https://doi.org/10.1101/2023.04.10.536199; this version posted April 10, 2023. The copyright holder for this preprint (which was not certified by peer review) is the author/funder, who has granted bioRxiv a license to display the preprint in perpetuity. It is made available under aCC-BY 4.0 International license.

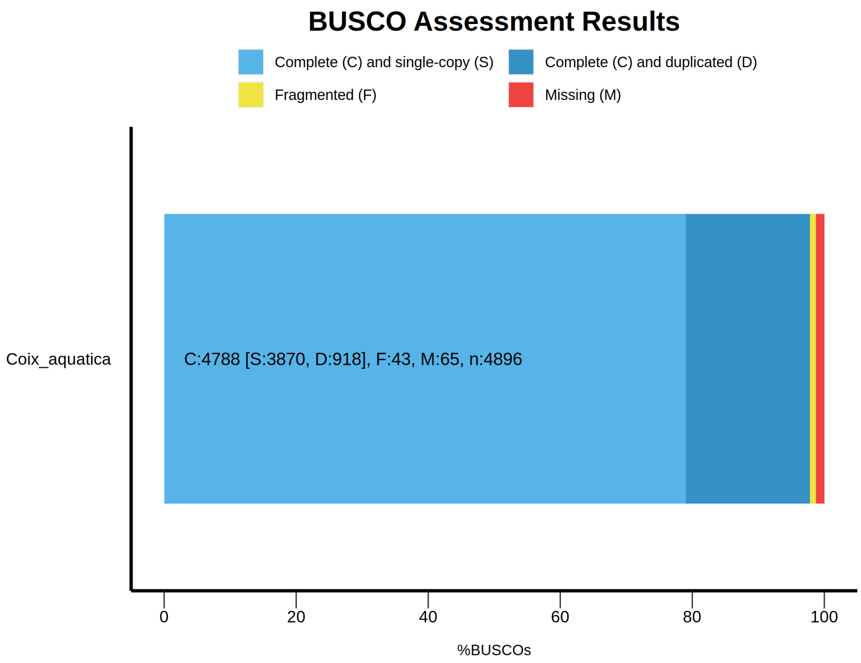

Figure S4: BUSCO scores (obtained with poales_odb10) of proteins predicted with GALBA in *Coix aquatica*.

Table S1: Donor proteins used for annotating each species genome with GALBA, FunAnnotate, and BRAKER2. Note: The proteins for whales and dolphins were applied to all whale and dolphin species with GALBA. *) Proteins were not used in the combined set but only for single protein set input experiments. ^{s}) Proteins were used to demonstrate GALBA accuracy with reference proteins from this species, alone (GALBA^{s} in Table 3).

**Species**	**Reference Protein File**
Arabidopsis thaliana	
Arabidopsis lyrata subsp. lyrata^{s}	GCF_000004255.2_v.1.0_protein.faa.gz
Arabidopsis thaliana x Arabidopsis arenosa	GCA_019202795.1_ASM1920279v1_protein.faa.gz
Camelina sativa	GCF_000633955.1_Cs_protein.faa.gz
Arabidopsis suecica	GCA_019202805.1_ASM1920280v1_protein.faa.gz
Capsella rubella	GCF_000375325.1_Caprub1_0_protein.faa.gz
Bombus terrestris	
Bombus vancouverensis nearcticus	GCF_011952275.1_Bvanc_JDL1245_protein.faa.gz
Bombus huntii	GCF_024542735.1_iyBomHunt1.1_protein.faa.gz
Bombus affinis	GCF_024516045.1_iyBomAffi1.2_protein.faa.gz
Bombus pyrosoma	GCF_014825855.1_ASM1482585v1_protein.faa.gz
Bombus vosnesenskii	GCF_011952255.1_Bvos_JDL3184-5_v1.1_protein.faa.gz
Bombus bifarius	GCF_011952205.1_Bbif_JDL3187_protein.faa.gz
Bombus impatiens^{s}	GCF_000188095.3_BIMP_2.2_protein.faa.gz
Caenorhabditis elegans	
Caenorhabditis auriculariae	GCA_904845305.1_CAUJ_protein.faa.gz
Caenorhabditis bovis	GCA_902829315.1_CBOVIS_v1.1_protein.faa.gz
Caenorhabditis brenneri	GCA_000143925.2_C_brenneri-6.0.1b_protein.faa.gz
Caenorhabditis briggsae^{s}	GCF_000004555.2_CB4_protein.faa.gz
Caenorhabditis remanei	GCF_000149515.1_ASM14951v1_protein.faa.gz
Danio rerio	
Cyprinus carpio	GCF_018340385.1_ASM1834038v1_protein.faa.gz
Carassius auratus	GCF_003368295.1_ASM336829v1_protein.faa.gz
Puntigrus tetrazona	GCF_018831695.1_ASM1883169v1_protein.faa.gz
Sinocyclocheilus rhinocerous	GCF_001515625.1_SAMN03320098_v1.1_protein.faa.gz
Sinocyclocheilus anshuiensis	GCF_001515605.1_SAMN03320099.WGS_v1.1_protein.faa.gz
Onychostoma macrolepis^{s}	GCA_012432095.1_ASM1243209v1_protein.faa.gz
Carassius gibelio	GCF_023724105.1_carGib1.2-hapl.c_protein.faa.gz
Pimephales promelas	GCF_016745375.1_EPA_FHM_2.0_protein.faa.gz
Labeo rohita	GCF_022985175.1_IGBB_LRoh.1.0_protein.faa.gz
Megalobrama amblycephala	GCF_018812025.1_ASM1881202v1_protein.faa.gz

bioRxiv preprint doi: https://doi.org/10.1101/2023.04.10.536199; this version posted April 10, 2023. The copyright holder for this preprint (which was not certified by peer review) is the author/funder, who has granted bioRxiv a license to display the preprint in perpetuity. It is made available under aCC-BY 4.0 International license.

Sinocyclocheilus grahami	GCF_001515645.1_SAMN03320097.WGS_v1.1_protein.faa.gz
Ctenopharyngodon idella	GCF_019924925.1_HZGC01_protein.faa.gz
Drosophila melanogaster	
Drosophila ananassae^s	GCF_017639315.1_ASM1763931v2_protein.faa.gz
Drosophila erectra*	GCF_003286155.1_DereRS2_protein.faa.gz
Drosophila grimshawi	GCF_018153295.1_ASM1815329v1_protein.faa.gz
Drosophila pseudoobscura	GCF_009870125.1_UCI_Dpse_MV25_protein.faa.gz
Drosophila simulans*	GCF_016746395.2_Prin_Dsim_3.1_protein.faa.gz
Drosophila virilis	GCF_003285735.1_DvirRS2_protein.faa.gz
Drosophila willistoni	GCF_018902025.1_UCI_dwil_1.1_protein.faa.gz
Musca domestica*	GCF_000371365.1_Musca_domestica-2.0.2_protein.faa.gz
Gallus gallus	
Lagopus muta	GCF_023343835.1_bLagMut1_primary_protein.faa.gz
Tympanuchus pallidicinctus	GCF_026119805.1_pur_lepc_1.0_protein.faa.gz
Lagopus leucura	GCF_019238085.1_USGS_WTPT01_protein.faa.gz
Centrocercus urophasianus	GCF_019232065.1_USGS_Curo_1.0_protein.faa.gz
Centrocercus urophasianus	GCF_019232065.1_USGS_Curo_1.0_protein.faa.gz
Coturnix japonica^s	GCF_001577835.2_Coturnix_japonica_2.1_protein.faa.gz
Meleagris gallopavo	GCF_000146605.3_Turkey_5.1_protein.faa.gz
Medicago truncatula	
Trifolium pratense^s	GCF_020283565.1_ARS_RC_1.1_protein.faa.gz
Pisum sativum	GCF_024323335.1_CAAS_Psat_ZW6_1.0_protein.faa.gz
Cicer arietinum	GCF_000331145.1_ASM33114v1_protein.faa.gz
Mus musculus	
Arvicanthis niloticus	GCF_011762505.1_mArvNil1.pat.X_protein.faa.gz
Grammomys surdaster	GCF_004785775.1_NIH_TR_1.0_protein.faa.gz
Mastomys coucha	GCF_008632895.1_UCSF_Mcou_1_protein.faa.gz
Mus pahari	GCF_900095145.1_PAHARI_EIJ_v1.1_protein.faa.gz
Apodemus sylvaticus	GCF_947179515.1_mApoSyl1.1_protein.faa.gz
Mus caroli^s	GCF_900094665.1_CAROLI_EIJ_v1.1_protein.faa.gz
Rattus rattus	GCF_011064425.1_Rrattus_CSIRO_v1_protein.faa.gz
Rattus norvegicus	GCF_015227675.2_mRatBN7.2_protein.faa.gz
Homo sapiens	GCF_000001405.40_GRCh38.p14_protein.faa.gz
Parasteatoda tepidariorum	
Trichonephila inaurata	GCA_019973955.1_Tnin_1.0_protein.faa.gz
Caerostris extrusa	GCA_021605095.1_Cext_1.0_protein.faa.gz
Caerostris darwini	GCA_021605075.1_Cdar_1.0_protein.faa.gz
Oedothorax gibbosus	GCA_019343175.1_Ogib_1.0_protein.faa.gz
Trichonephila clavata	GCA_019973975.1_Tnct_1.0_protein.faa.gz
Trichonephila clavipes	GCA_019973935.1_Tncv_1.0_protein.faa.gz
Araneus ventricosus^s	GCA_013235015.1_Ave_3.0_protein.faa.gz
Nephila pilipes	GCA_019974015.1_Npil_1.0_protein.faa.gz
Rhodnius prolixus	
Nesidiocoris tenuis	GCA_902806785.1_CYROTEf_10X_genome_protein.faa.gz
Cimex lectularius^s	GCF_000648675.2_Clec_2.1_protein.faa.gz
Halyomorpha halys	GCF_000696795.2_Hhal_2.0_protein.faa.gz
Nezara viridula	GCA_928085145.1_PGI_NEZAVIv3_protein.faa.gz
Populus trichocarpa	
Populus tomentosa	GCA_018804465.1_PTv2_protein.faa.gz
Populus euphratica	GCF_000495115.1_PopEup_1.0_protein.faa.gz
Populus alba	GCF_005239225.1_ASM523922v1_protein.faa.gz
Populus deltoides^s	GCA_015852605.2_ASM1585260v2_protein.faa.gz
Solanum lycopersicum	
Solanum stenotomum	GCF_019186545.1_ASM1918654v1_protein.faa.gz
Solanum tuberosum	GCF_000226075.1_SolTub_3.0_protein.faa.gz
Solanum verrucosum	GCF_900185275.1_falcon-dt-bn_protein.faa.gz
Solanum pennellii^s	GCF_001406875.1_SPENNV200_protein.faa.gz
Tetraodon nigroviridis	
Micropterus salmoides	GCF_014851395.1_ASM1485139v1_protein.faa.gz
Gasterosteus aculeatus aculeatus	GCF_016920845.1_GAculeatus_UGA_version5_protein.faa.gz
Sebastes umbrosus	GCF_015220745.1_fSebUmb1.pri_protein.faa.gz
Etheostoma cragini	GCF_013103735.1_CSU_Ecrag_1.0_protein.faa.gz
Gymnodraco acuticeps	GCF_902827175.1_fGymAcu1.1_protein.faa.gz
Pseudochaenichthys georgianus	GCF_902827115.1_fPseGeo1.1_protein.faa.gz
Dissostichus mawsoni	GCA_011823955.1_KU_Dm_1.0_protein.faa.gz
Cyclopterus lumpus	GCF_009769545.1_fCycLum1.pri_protein.faa.gz
Notolabrus celidotus	GCF_009762535.1_fNotCel1.pri_protein.faa.gz

bioRxiv preprint doi: https://doi.org/10.1101/2023.04.10.536199; this version posted April 10, 2023. The copyright holder for this preprint (which was not certified by peer review) is the author/funder, who has granted bioRxiv a license to display the preprint in perpetuity. It is made available under aCC-BY 4.0 International license.

Etheostoma spectabile	GCF_008692095.1_UIUC_Espe_1.0_protein.faa.gz
Anarrhichthys ocellatus	GCF_004355925.1_GSC_Weel_1.0_protein.faa.gz
Cottoperca gobio	GCF_900634415.1_fCotGob3.1_protein.faa.gz
Takifugu rubripes[s]	GCF_901000725.2_fTakRub1.2_protein.faa.gz
Xenopus tropicalis	
Xenopus laevis[s]	GCF_001663975.1_Xenopus_laevis_v2_protein.faa.gz
Hymenochirus boettgeri	GCA_019447015.1_UCB_Hboe_1.0_protein.faa.gz
Eleutherodactylus coqui	GCA_019857665.1_UCB_Ecoq_1.0_protein.faa.gz
Engystomops pustulosus	GCA_019512145.1_UCB_Epus_1.0_protein.faa.gz
Bufo bufo	GCF_905171765.1_aBufBuf1.1_protein.faa.gz
Spea bombifrons	GCF_027358695.1_aSpeBom1.2.pri_protein.faa.gz
Rana temporaria	GCF_905171775.1_aRanTem1.1_protein.faa.gz
Bufo gargarizans	GCF_014858855.1_ASM1485885v1_protein.faa.gz
Bombina bombina	GCF_027579735.1_aBomBom1.pri_protein.faa.gz
Wales and dolphins	
Lipotes vexillife	GCF_000442215.2_Lipotes_vexillifer_v1.1_protein.faa.gz
Delphinapterus leucas	GCF_002288925.2_ASM228892v3_protein.faa.gz
Monodon monoceros	GCF_005190385.1_NGI_Narwhal_1_protein.faa.gz
Tursiops truncatus	GCF_011762595.1_mTurTru1.mat.Y_protein.faa.gz
Neophocaena asiaeorientalis	GCF_003031525.2_Neophocaena_asiaeorientalis_V1.1_protein.faa.gz
Phocoena sinus	GCF_008692025.1_mPhoSin1.pri_protein.faa.gz
Lagenorhynchus obliquidens	GCF_003676395.1_ASM367639v1_protein.faa.gz
Pontoporia blainvillei	GCA_011754075.1_ASM1175407v1_protein.faa.gz
Globicephala melas	GCF_006547405.1_ASM654740v1_protein.faa.gz
Orcinus orca	GCF_937001465.1_mOrcOrc1.1_protein.faa.gz
Physeter catodon	GCF_002837175.2_ASM283717v2_protein.faa.gz
Coix aquatica	
Zea mays	GCF_902167145.1_Zm-B73-REFERENCE-NAM-5.0_protein.faa.gz
Sorghum bicolor	GCF_000003195.3_Sorghum_bicolor_NCBIv3_protein.faa.gz
Miscanthus lutarioriparius	GCA_904845875.1_Mlu_assembly_protein.faa.gz
Panicum hallii	GCF_002211085.1_PHallii_v3.1_protein.faa.gz

	miniprot raw				miniprothint all				miniprothint HC			
	TP	FP	Sn	Sp	TP	FP	Sn	Sp	TP	FP	Sn	Sp
five close relatives	38,342	9,612	80.3	80.0	37,639	4,230	78.8	89.9	32,896	511	68.9	98.5
ODB order excluded	29,640	390,978	62.1	7.1	25,427	82,094	53.3	23.7	18,315	1,878	38.4	90.7

Table S2: Comparison of intron predictions by spliced alignment using a protein set of closely related species (see Table S1), and the OrthoDB v.11 (ODB) Arthopoda partition (proteins from species of the same order excluded) on *D. melanogaster*. The reference annotation has 47,739 introns. The values in the table—True Positives (TP), False Positives (FP), Sensitivity (Sn), Specificity (Sp)—are shown for the raw miniprot result, all miniprothint predictions, and high-confidence (HC) miniprothint predictions (see Figure 3 for details).

bioRxiv preprint doi: https://doi.org/10.1101/2023.04.10.536199; this version posted April 10, 2023. The copyright holder for this preprint (which was not certified by peer review) is the author/funder, who has granted bioRxiv a license to display the preprint in perpetuity. It is made available under aCC-BY 4.0 International license.

	Gene Sensitivity		Exon Sensitivity	
	GALBA	BRAKER2	GALBA	BRAKER2
A. thaliana	86.76	**91.22**	89.97	**91.02**
B. terrestris	**78.31**	76.03	**89.74**	86.98
C. elegans	59.56	**77.03**	80.59	**88.30**
D. melanogaster	72.43	**77.73**	81.43	**82.16**
M. truncatula	62.40	**69.19**	88.56	**91.63**
P. tepidariorum	44.92	**45.26**	81.19	**82.02**
P. trichocarpa	75.80	**83.51**	90.59	**92.41**
R. prolixus	42.25	**47.90**	77.37	**81.48**
S. lycopersicum	75.88	**77.17**	94.02	**94.55**
T. nigroviridis	**71.12**	**71.12**	**91.91**	90.61
X. tropicalis	**72.21**	54.95	**91.45**	83.97

Table S3: Feature prediction Sensitivity in a subset of annotated multi-exon genes that have support by spliced RNA-Seq to genome alignments in all introns.

	Gene Sensitivity		Exon Sensitivity	
	GALBA	BRAKER2	GALBA	BRAKER2
D. rerio	**70.16**	58.78	**93.49**	89.4
G. gallus	**72.00**	30.16	**94.08**	37.61
M. musculus	**77.85**	40.31	**95.18**	61.38

Table S4: Feature prediction Sensitivity in a subset of reliably annotated genes. A gene is regarded as reliable if a minimum of two annotation sets contain this exact gene structure.

bioRxiv preprint doi: https://doi.org/10.1101/2023.04.10.536199; this version posted April 10, 2023. The copyright holder for this preprint (which was not certified by peer review) is the author/funder, who has granted bioRxiv a license to display the preprint in perpetuity. It is made available under aCC-BY 4.0 International license.

Tool	Version (or commit)
GALBA	1.0.6
Python	3.8
miniprot	0.9-r224-dirty
augustus	3.5.0
miniprothint	a38f300
miniprot-boundary-scorer	37493bc
braker.pl	3.0.0
TSEBRA	b0d6c4f
GeneMark-EP/ETP	ede6bc5
BUSCO	5.4.2
FunAnnotate	v1.8.14
Exonerate	v2.4.0
DIAMOND	v2.0.15
EvidenceModeler	1.1.1
GeneMark (FunAnnotate)	v4.71_lic
tbl2asn	25.8
bedtools	v2.30.0
augustus (FunAnnotate)	3.3.2
tRNAscan-SE	2.0.9
minimap2	2.24-r1122
RepeatModeler	2.0.4
RepeatMasker	4.1.4
NCBI/RMBLAST	2.13.0+
TRF	4.09
RECON	1.08
RepeatScout	1.0.5
GenomeTools	1.6.0
LTR_Retriever	v2.9.0
Ninja	0.97
MAFFT	7.471
CD-HIT	4.8.1
Singularity	3.10.0-dirty

Table S5: Software versions.

bioRxiv preprint doi: https://doi.org/10.1101/2023.04.10.536199; this version posted April 10, 2023. The copyright holder for this preprint (which was not certified by peer review) is the author/funder, who has granted bioRxiv a license to display the preprint in perpetuity. It is made available under aCC-BY 4.0 International license.

Species	BUSCO seed species	BUSCO DB
Arabidopsis thaliana	cacao	embryophyta
Bombus terrestris	fly	arthropoda
Caenorhabditis elegans	trichinella	metazoa
Dano rerio	human	vertebrata
Drosophila melanogaster	nasonia	arthropoda
Gallus gallus	human	tetrapoda
Medicago truncatula	cacao	embryophyta
Mus musculus	chicken	tetrapoda
Parasteatoda tepdariorum	fly	arthropoda
Populus trichocarpa	cacao	embryophyta
Rhodnius prolixus	fly	arthropoda
Solanum lycopersicum	cacao	embryophyta
Tetraodon nigroviridis	human	vertebrata
Xenopus tropicalis	human	tetrapoda

Table S6: Seed species and BUSCO DB used for BUSCO with FunAnnotate. Parameters were selected in such a way that the species that the AUGUSTUS parameters were trained on is not part of the same order as the target species. We use this scenario to simulate what will happen when annotating representatives of novel clades.

	Arabidopsis thaliana			*Bombus terrestris*			*Caenorhabditis elegans*			*Danio rerio*			*Drosophila melanogaster*		
	Gene	Transcript	Exon	Gene	Transcript	Exon	Gene	Transcript	Exon	Gene	Transcript	Exon	Gene	Transcript	Exon
BRAKER2 ODB+	76.95	61.46	85.11	47.41	39.23	79.58	69.31	56.27	87.90	29.89	23.59	72.86	76.80	58.68	83.88
BRAKER2 ODB^o	71.17	56.33	83.97	37.32	29.42	75.49	51.30	41.62	80.48	27.20	21.82	72.15	60.61	46.03	76.66
FunAnnotate	77.26	61.81	87.03	35.51	29.04	71.66	45.53	37.39	77.84	8.95	7.40	47.04	58.24	44.68	74.41
	Medicago truncatula			*Parasteatoda tepidariorum*			*Populus trichocarpa*			*Rhodnius prolixus*			*Tetraodon nigroviridis*		
	Gene	Transcript	Exon	Gene	Transcript	Exon	Gene	Transcript	Exon	Gene	Transcript	Exon	Gene	Transcript	Exon
BRAKER2 ODB+	46.93	45.06	74.82	21.48	19.08	64.09	66.13	57.20	82.95	13.35	12.83	54.88	9.39	8.24	58.56
BRAKER2 ODB^o	44.80	43.52	74.76	19.33	17.36	62.60	63.65	55.09	82.61	12.77	12.41	54.38	9.21	8.20	58.47
FunAnnotate	33.33	33.33	67.89	13.71	12.48	55.20	50.11	44.38	75.94	6.89	6.89	29.51	4.42	4.11	36.91
	Gallus gallus			*Mus musculus*			*Solanum lycopersicum*			*Xenopus tropicalis*			**Average**		
	Gene	Transcript	Exon	Gene	Transcript	Exon	Gene	Transcript	Exon	Gene	Transcript	Exon	Gene	Transcript	Exon
BRAKER2 ODB+	23.11	15.60	45.57	27.20	16.90	57.27	38.45	36.12	69.41	36.48	28.23	78.21	41.63	34.18	71.08
BRAKER2 ODB^o	20.14	18.53	42.83	27.01	26.41	66.09	37.50	36.29	71.29	31.18	23.97	75.91	36.66	31.22	69.78
FunAnnotate	15.4	10.05	44.21	NA	NA	NA	31.94	31.94	66.28	NA	NA	NA	NA	NA	NA

Table S7: F1-scores of gene predictions from BRAKER2 executed with OrthoDB v11 partitions (species excluded) and proteins of closely related species (BRAKER2 ODB+), and BRAKER2 results with OrthoDB v11 partitions where proteins from the same order as the target species have been excluded (BRAKER2 ODB^o), and results of FunAnnotate. FunAnnotate went out of memory for *M. musculus* and *X. tropicalis* on our HPC nodes that had 189 GB RAM.

Species	Single	Duplicated	Duplicated, Expected	Duplicated, Unexpected	Missing	Consistent	Consistent, partial hits	Consistent, fragmented hits	Inconsistent	Inconsistent, partial hits	Inconsistent fragmented	Contaminants	Unknown
Balaenoptera bonaerensis	54.48	44.28	43.47	0.81	1.23	71.10	11.13	41.53	28.52	5.27	21.69	0	0.39
Eubalanea japonica	67.59	31.03	30.25	0.77	1.39	67.91	9.08	32.42	31.78	4.26	23.26	0	0.31
Inia geoffrensis	69.99	27.92	27.34	0.58	2.08	69.99	9.16	31.67	29.75	4.10	21.56	0	0.26
Kogia previceps	63.27	35.13	34.66	0.48	1.59	67.60	10.14	35.59	32.19	4.87	23.90	0	0.22
Phocoena phocoena	76.97	21.48	20.77	0.70	1.55	67.44	8.11	28.47	32.21	4.71	23.76	0	0.35
Platanista gangetica	54.51	44.30	43.62	0.67	1.19	70.54	11.59	37.75	29.11	4.51	21.66	0	0.34
Ziphius cavirostris	48.30	49.67	49.12	0.57	2.02	74.62	13.19	44.83	25.18	4.13	18.42	0	0.20
Coix aquatica	85.25	10.98	8.59	2.39	3.67	48.01	6.81	9.88	49.27	7.61	32.42	0	2.72

Table S8: OMArk results (in percent) in genomes that were *de novo* annotated with GALBA. The number of conserved HOGs for whales and dolphins is 13,050, the number of conserved HOGs for *Coix aquatica* is 20,501.

bioRxiv preprint doi: https://doi.org/10.1101/2023.04.10.536199; this version posted April 10, 2023. The copyright holder for this preprint (which was not certified by peer review) is the author/funder, who has granted bioRxiv a license to display the preprint in perpetuity. It is made available under aCC-BY 4.0 International license.

OrthoDB partition	Size (#sequences)	Test species
arthropoda_odb11	4,307,558	*Bombus terrestris*, *Drosophila melanogaster*, *Parasteatoda tepidariorum*, *Rhodnius prolixus*
metazoa_odb11	15,257,394	*Caenorhabditis elegans*
vertebrata_odb11	9,805,833	*Danio rerio*, *Gallus gallus*, *Tetraodon nigroviridis*, *Mus musculus*
viridiplantae_odb11	5,310,477	*Arabidopsis thaliana*, *Medicago truncatula*, *Populus trichocarpa*, *Solanum lycopersicum*

Table S9: Overview of the OrthoDB partitions and the test species for which they were used. For results in Table 1, each test species, species belonging to the same taxonomic order were excluded from the databases for each experiment. We used the orthodb-clades pipeline to generate the protein sets. For results in Table S7, only the target species were excluded, and this ODB partition was subsequently combined with the close relatives input from Table S1 by concatenation prior to execution of BRAKER2.

bioRxiv preprint doi: https://doi.org/10.1101/2023.04.10.536199; this version posted April 10, 2023. The copyright holder for this preprint (which was not certified by peer review) is the author/funder, who has granted bioRxiv a license to display the preprint in perpetuity. It is made available under aCC-BY 4.0 International license.

Supplementary Methods

Assembly Quality Estimation

We used seqstats from `https://github.com/clwgg/seqstats` to compute genome sizes, (scaffold) N50, and the total number of sequences.

Annotation Parameter Computation

In order to count genes and alternative transcripts thereof, we renamed the genes and transcripts in reference annotations with the script `rename_gtf.py` from `https://github.com/Gaius-Augustus/TSEBRA` as follows:

```
rename_gtf.py --gtf annot.gtf --out annot_tsebra.out
```

Subsequently, we extracted the last gene id as number of genes, and computed the number of transcripts:

```
cat annot_tsebra.gtf | perl -ne ' \
     if(m/transcript_id \"([^"]+)\"/){print $1."\n";}'| sort -u | wc -l
```

The ratio of mono-exonic to multi-exonic genes was computed with `analyze_exons.py` from `https://github.com/Gaius-Augustus/GALBA`:

```
analyze_exons.py -f file.gtf
```

In case of RNA-Seq supported 'reliable' genes, the number was computed with `complete_supported_subset_table.sh` from `https://github.com/gatech-genemark/BRAKER2-exp`:

```
complete_supported_subset_table.sh prediction.gtf annot.gtf completeTranscripts.gtf \
    pseudo.gff3 varus.gff
```

Running FunAnnotate

FunAnnotate was executed from a singularity container as follows:

```
# only once, to get the singularity container
singularity pull docker://nextgenusfs/funannotate

export GENEMARK_PATH=/path/to/GeneMark-ES-ET-EP_v4.71_lic/gmes_funannotate

species="name of species"
buscoSeedSpecies="name of seed species"
buscodb="name of busco db"
genomepath="/path/to/genome.fasta.masked"
protpath="/path/to/proteins.fa"

# calculateGenomeSizeFromFasta.pl adds up the length of all sequences in a fasta
genomeSize=$(perl ~/calculateGenomeSizeFromFasta.pl $genomepath)
maxIntronLen_f=$(echo "3.6 * sqrt($genomeSize)" | bc -l)
maxIntronLen=$(printf "%.0f" "$maxIntronLen_f")

mkdir -p fun tmp
singularity run funannotate_latest.sif funannotate predict \
    --input $genomepath --out fun --species $species \
    --busco_seed_species $buscoSeedSpecies --busco_db $buscodb \
    --organism other --protein_evidence $protpath \
    --max_intronlen $maxIntronLen --cpus 72 --tmpdir tmp --no-progress \
    --repeats2evm
```

bioRxiv preprint doi: https://doi.org/10.1101/2023.04.10.536199; this version posted April 10, 2023. The copyright holder for this preprint (which was not certified by peer review) is the author/funder, who has granted bioRxiv a license to display the preprint in perpetuity. It is made available under aCC-BY 4.0 International license.

For accuracy evaluation, the gff3 output of FunAnnotate was converted from gff3 to gtf format using `gff3_to_gtf.pl` from GeneMark-ET, and with `compute_accuracies.sh` from BRAKER:

```
gff3_to_gtf.pl funannotate.gff3 funannotate.gtf
compute_accuracies.sh annot.gtf pseudo.gff3 funannotate.gtf gene trans cds
```

FunAnnotate sometimes modifies sequence names in the output, automatically. We had to revert these sequence name changes to match the reference annotation. This was in particular the case for *Medicago truncatula*:

```
cat funannotate.gtf | perl -pe 's/Mrun/Mtrun/' > funannotate.f.gtf
mv funannotate.f.gtf funannotate.gtf
```

Running GALBA

GALBA was executed as follows:

```
galba.pl --genome=genome.fa --prot_seq=proteins.fa --threads 72
```

The number of threads varied between runs, depending on HPC node availability.

Running BRAKER2

BRAKER2 was executed with singularity as follows:

```
singularity exec braker3.sif braker.pl --genome=genome.fa --prot_seq=proteins.fa --threads 72
```

The number of threads varied between runs, depending on HPC node availability.

Running TSEBRA

TSEBRA was executed as follows:

```
tsebra.py -g braker.gtf --keep_gtf galba.gtf \
    -e braker_hintsfile.gff,galba_hintsfile.gff -c default.cfg -o tsebra.gtf
```
